# Progress and challenges in bioinformatics approaches for enhancer identification

**DOI:** 10.1093/bib/bbv101

**Published:** 2015-12-03

**Authors:** Dimitrios Kleftogiannis, Panos Kalnis, Vladimir B. Bajic

**Keywords:** gene regulation, enhancers, chromatin signatures, histone modification marks, genome annotation, machine learning, bioinformatics, computer science

## Abstract

Enhancers are *cis*-acting DNA elements that play critical roles in distal regulation of gene expression. Identifying enhancers is an important step for understanding distinct gene expression programs that may reflect normal and pathogenic cellular conditions. Experimental identification of enhancers is constrained by the set of conditions used in the experiment. This requires multiple experiments to identify enhancers, as they can be active under specific cellular conditions but not in different cell types/tissues or cellular states. This has opened prospects for computational prediction methods that can be used for high-throughput identification of putative enhancers to complement experimental approaches. Potential functions and properties of predicted enhancers have been catalogued and summarized in several enhancer-oriented databases. Because the current methods for the computational prediction of enhancers produce significantly different enhancer predictions, it will be beneficial for the research community to have an overview of the strategies and solutions developed in this field. In this review, we focus on the identification and analysis of enhancers by bioinformatics approaches. First, we describe a general framework for computational identification of enhancers, present relevant data types and discuss possible computational solutions. Next, we cover over 30 existing computational enhancer identification methods that were developed since 2000. Our review highlights advantages, limitations and potentials, while suggesting pragmatic guidelines for development of more efficient computational enhancer prediction methods. Finally, we discuss challenges and open problems of this topic, which require further consideration.

## Introduction

Gene expression in eukaryotes is governed by complex processes orchestrated by the interplay of various elements located in DNA regulatory regions [1–4]. Enhancers represent one of the better-characterized regulatory elements. Enhancers increase the transcriptional output in cells manifesting distinct properties, which are summarized as follows [5–8]: (a) enhancers reside thousands of base pairs upstream or downstream from the transcription start sites (TSSs) of their target genes or they can even be on different chromosomes relative to their targets, (b) they may exhibit tissue-specific properties and (c) they may initiate RNA polymerase II transcription, producing a new class of non-coding RNAs called enhancer RNAs (eRNAs).

Previous gene regulation studies have emphasized the role of enhancers in transcription initiation [9]. Analysis of enhancer properties has also raised key questions about mechanisms that govern the fate of temporal and tissue-specific gene expression. In addition, several studies [10, 11] have linked variations in enhancer sequences to cancer and other diseases. In particular, identifying enhancers and understanding their mechanisms of functioning is an area of great interest that may enrich our current knowledge about diseases and therapeutic strategies [12, 13].

So far, some review articles have focused on different aspects of enhancer functions that characterize cell identity or pathogenic states [14, 15]. In addition, the enhancer mechanistic properties aimed at identifying active enhancers are well documented in several studies and reviews, including advances in high-throughput experimental technologies [16–18]. However, because active enhancers are characterized by specific cellular properties and because there are numerous cellular conditions, experimental identification of enhancers faces certain limitations [17]. For this reason, computational identification of enhancers has been well studied in recent years and has resulted in a number of computational methods that complement the experimental techniques [19, 20]. Moreover, the generation of new types of high-throughput data helped to improve prediction models for enhancers. However, despite the efforts to develop accurate enhancer prediction methods [21–55], the current solutions generate significantly different enhancer predictions. In Table 1, we present the pairwise intersection of enhancer predictions as obtained in [50] by five state-of-the-art methods across six ENCODE (Encyclopedia of DNA Elements) cell lines. It is apparent that the overlap of computationally predicted sets of enhancers is relatively small. Consequently, it will be beneficial for the research community to have an overview of the strategies and solutions developed in this field.

**Table 1:** Comparison analysis of enhancer predictions obtained by different methods across six ENCODE cell lines

Method 1 versus Method 2	Gm12878			H1hesc			K562			HeLa			HepG2			HUVEC		
	**Coverage 1 versus. Coverage 2 (million bases)**	**Overlap (million bases)**	**Jaccard inde× (%)**	**Coverage 1 versus Coverage 2 (million bases)**	**Overlap (million bases)**	**Jaccard inde× (%)**	**Coverage 1 versus Coverage 2 (million bases)**	**Overlap (million bases)**	**Jaccard inde× (%)**	**Coverage 1 versus Coverage 2 (million bases)**	**Overlap (million bases)**	**Jaccard index (%)**	**Coverage 1 versus Coverage 2 (million bases)**	**Overlap (million bases)**	**Jaccard inde× (%)**	**Coverage 1 versus Coverage 2 (million bases)**	**Overlap (million bases)**	**Jaccard index (%)**
**CSI-ANN versus ENCODE annotation**	10.7 versus 42.69	5.3	11.0	19.1 versus 56.5	2.2	3.0	34.5 versus 28.1	8.0	14.8	26.6. versus 42.9	5.3	8.3	40.8 versus 24	6.4	11.0	49.4 versus 47.1	21.3	28.3
**RFECS versus ENCODE annotation**	344.1 versus 42.69	31.6	8.9	124.7 versus 56.5	29.5	19.4	130.6 versus 28.1	18.5	13.1	87.4 versus 42.9	26.4	25.4	253.1 versus 24	12	4.5	191.2 versus 47.1	33.6	16.4
**ChromHMM versus ENCODE annotation**	82.7 versus 42.69	37.7	43.0	80.5 versus 56.5	36.9	**36.8**	111.4 versus. 28.1	24.8	21.6	70.9 versus 42.9	36.0	**46.2**	72.88 versus 24	10.8	12.6	107.2 versus 47.1	40.0	35.0
**Segway versus ENCODE annotation**	119.5 versus 42.69	39.1	31.7	404.9 versus 56.5	20.3	4.6	282.5 versus 28.1	27.6	9.7	124.8 versus 42.9	41.1	32.7	230.3 versus 24	11.6	4.5	189.6 s. 47.1	39.1	19.7
CSI-ANN versusRFECS	10.7 versus 344.1	6.1	1.7	19.1 versus 124.7	2.2	1.5	34.5 versus 130.6	11.4	7.4	26.6 versus 87.4	5.71	5.2	40.8 versus 253.1	12.6	4.4	49.4 versus 191.2	21.9	10.0
RFECS versus ChromHMM	344.1 versus 82.7	55.9	15.0	124.7 versus 80.5	40.3	24.4	34.5 versus 111.4	51.1	26.7	87.4 versus 70.9	42.1	36.1	253.1 versus 72.88	45.2	16.2	191.2 versus 107.2	73.7	32.7
RFECS versus Segway	344.1 versus 119.5	71.6	18.2	124.7 versus 404.9	52.3	10.9	130.6 versus. 282.5	80.5	24.2	87.4 versus 124.8	51.1	31.7	253.1 versus 230.3	77.8	19.1	191.2 versus 189.6	92.4	32.0
CSI-ANN versus ChromHMM	10.7 versus 82.7	6.0	6.8	19.1 versus 80.5	1.6	1.6	34.5 versus 111.4	12.0	8.9	26.6 versus 70.9	5.8	6.3	40.8 versus 72.88	10.9	10.6	49.4 versus 107.2	25.8	19.7
ChromHMM versus Segway	82.7 versus 119.5	63.8	**46.1**	80.5 versus 404.9	52.5	12.1	111.4 versus 282.5	100.6	**34.2**	70.9 versus 124.8	61.4	45.7	72.88 versus 230.3	56.3	**22.8**	107.2 versus 189.6	99.0	**50.0**
CSI-ANN versus Segway	10.7 versus 119.5	8.0	6.5	19.1 versus 404.9	1.3	0.3	34.5 versus 282.5	19.74	6.6	26.6. versus 124.8	10.0	7.1	40.8 versus 230.3	18.1	7.1	49.4 versus 189.6	30.4	14.5

We report the total number of bases in millions predicted as belonging to enhancers. Coverage 1 corresponds to enhancers predicted by Method 1, while Coverage 2 corresponds to enhancers predicted by Method 2. The overlap column corresponds to the same enhancer predictions in million bases as obtained by Method 1 and Method 2. In the third column, we report similarity of predictions of Method 1 and Method 2 based on the Jaccard similarity index (as percentage)

With this issue in mind, we focused our efforts on bioinformatics approaches for enhancer identification published from 2000 to 2015, characterized by the use of data from high-throughput experiments for the development of enhancer prediction models. First, we present the basic principles of a general framework for enhancer identification. Next, we cover a comprehensive list of over 30 existing enhancer recognition tools and methods that have been developed in the considered period. Our aim is to analyse the existing approaches to provide useful comments regarding the data sets used and the prevalent computational solutions. In a separate section, we comment on obstacles that the existing methods face, address challenges and open questions related to enhancer identification and hint on promising directions for future research. Finally, we summarize available enhancer resources and suggest pragmatic guidelines for using the available computational solutions and relevant enhancer data.

## Computational identification of enhancers: the framework

**Figure 1:**
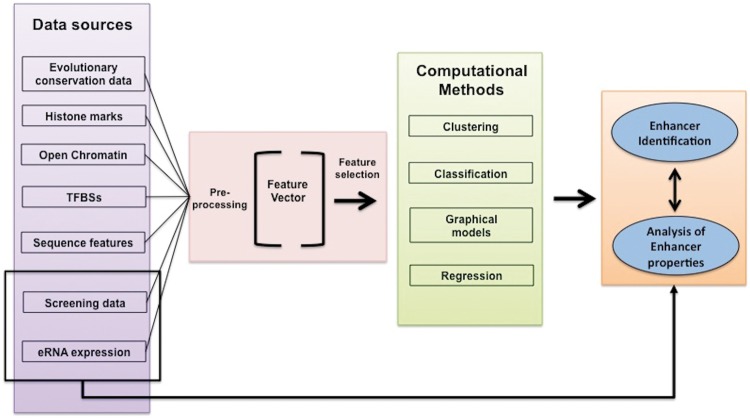
This figure shows basic components of a general enhancer identification system. The first block on the left (lille colour) handles integration and preprocessing of different data types. These data types (summarized in Table 2) can be combined in different ways to generate feature vectors that describe DNA regions. The feature values can be normalized or rescaled (second block-red colour). Then, FS techniques can be applied to reduce the number of features and select smaller sets of features with higher discriminative capabilities. The feature vectors feed computational models that make decisions using unsupervised and/or supervised algorithms (third block-green colour). Outcome is a list of identified enhancer regions (fourth block-orange colour), which can be analysed further using computational techniques.

The problem of computational identification of enhancers can be formulated as follows: ‘Given a DNA region described by multiple data types, determine if it can function as an enhancer'. Figure 1 depicts an overview of a general enhancer identification process.

The first step concerns integration of different data types coming from different data sources and preprocessing to generate feature vectors that serve as input for the enhancer identification and analysis system. The feature vectors contain information that describes data instances. Typically, these feature vectors capture information about evolutionary conservation [20] (e.g. regions or motifs that are highly conserved across different species), and/or chromatin profiles of histone marks as derived from ChIP-seq (chromatin immunoprecipitation with massively parallel DNA sequencing) data [28] and/or chromatin accessibility information as derived from DNase I hypersensitivity sites (DHS). The previous data types are frequently combined with transcription factor-binding sites (TFBSs) for identifying different classes of regulatory elements (e.g. enhancers, promoters, etc.) [25]. Note that with the acronym TFBSs, we refer to both the actual and the predicted DNA-binding sites of DNA-binding proteins that facilitate transcription, including transcription factors (TFs) and additional binding proteins or protein complexes such as the nucleosome remodelling complex (e.g. SWI/SNF), or histone acetyltransferases (HATs; e.g. P300 from HATs) and histone methyltransferases (HMTs; e.g. ASH1L from HMTs). Recently, enhancer-screening data, as well as expression of eRNAs, can serve as input for identifying enhancers and analysing their properties. In Table 2, we present an overview of the features used by different computational methods for enhancers’ identification. The process of generating feature vectors may include additional steps of normalization or rescaling of the feature values.

**Table 2:** Overview of data and features used for enhancer identification

Data sources	Feature example	Advantage	Disadvantage	Representative methods
Evolutionary conservation	Conserved motifs across species	Easy to compute	Insufficient information for predicting enhancer's tissue-specific activity	[20]
Histone marks	ChIP-seq from H3K4me1	Provides cell-line-/tissue-specific information that characterize enhancers and also different categories of enhancers (e.g. poised versus active)	Different cell lines/tissues are associated with different combination of histone marks	[21, 28, 33, 34]
TFBSs	ChIP-seq from P300	Provides cell-line-/tissue-specific information that characterize enhancers. High-resolution data for testing activity of enhancer-related TFs	Not available for many cell lines/tissues	[23, 29]
Open chromatin	DHS	High discriminative capacity when combined with other data types, e.g. P300-binding sites	Regions with enriched DHS activity do not necessarily correspond to enhancers	[25]
Sequence characteristics	Kmers of size 5	Easy to compute	Insufficient information for predicting enhancers’ activity across different tissues	[39, 51]
eRNA expression	CAGE data	High accuracy	eRNA regulation mechanisms are unknown, and not all of the enhancers are known to produce eRNAs	[40]
Enhancer-screening data	STARR-seq	High accuracy for testing enhancer activity	Not useful for *ab initio* discovery of enhancers	[42, 43, 52]

In the second step, different computational models use feature vectors to annotate DNA regions. The computational models are developed by computational methods, unsupervised or supervised, using the same feature vectors to describe the data. The methods used include state-of-the-art clustering algorithms such as K-means [21] or bi-clustering [24], probabilistic graphical models (PGMs) such as Hidden Markov Models (HMMs) [30] or Dynamic Bayesian Networks (DBNs) [31], regression models such as least absolute shrinkage and selection operator [53] and more advanced supervised classification systems, such as support vector machines (SVMs) [34], artificial neural networks (ANNs) [33], decision trees (DTs) [38] and random forests (RFs) [37]. The most important difference between supervised and unsupervised techniques is the fact that supervised methods require prior knowledge (e.g. some representative enhancers and when available, non-enhancer examples) for training. In contrast, this is not the case for unsupervised methods, where enhancer regions (and other regulatory elements in general) can be identified *ab initio* and without any prior knowledge. Unsupervised techniques rely strongly on some *ad hoc* rules for assigning regions to the class of enhancers, and thus their predictive abilities have some limitations. An example is identification of enhancers using only H3K4me1 profiles, which of course is correct, but is insufficient because there is no guarantee that they can characterize in the same way enhancers from different cell lines and tissues can.

The main outcome of an enhancer identification system is a catalogue of predicted enhancers. The identified enhancers can be further analysed computationally for their properties, deciphering their regulatory roles and associating them with target genes and eRNAs.

**Figure 2:**
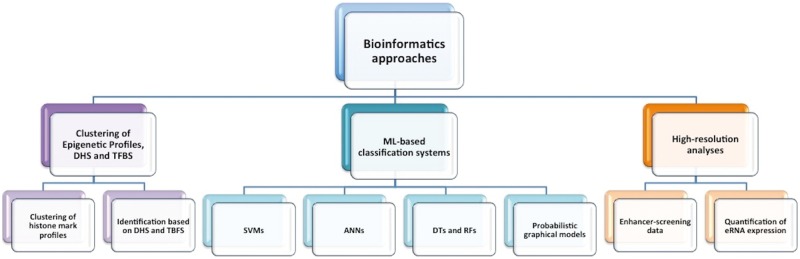
The figure presents the roadmap of existing approaches for enhancer identification. We have categorized the methods into three basic streams, which we partitioned further into subcategories based on the underlying computational solutions and the combination of relevant enhancer data.

A conceptually simple way to classify enhancer identification methods can be based on the available data sources (e.g. grouping together all methods that rely on evolutionary conservation). However, this is not readily applicable because different methods rely on a mixture of different data sets/features, and frequently the deployed algorithms combine supervised and unsupervised components. In this review, we group the available methods into three categories. The first category includes computational methods that identify DNA regulatory elements (including enhancers) using epigenetic signatures such as ChiP-seq of histone marks, DHS peaks and/or TFBSs mainly through unsupervised learning and clustering techniques [21–29]. The second category represents systems based on supervised machine learning (ML) classification that use mainly ChIP-seq data of histone marks frequently combined with sequence motifs, to distinguish enhancers from non-enhancers and identify features that characterize enhancers in an optimized way [33–39]. In this category, we also cover methods based on PGMs that are in the group of supervised learning methods [30–32]. As the third category, we consider recent bioinformatics methods that identify enhancers using as input experimental enhancer-screening data and data from some more targeted experiments. Although these methods are in principle experimental, the analysis of the results relies strongly on advanced bioinformatics methods combined with ML algorithms for deciphering the enhancer context [42–49]. Figure 2 gives the outline of existing bioinformatics approaches for enhancer identification. In Table 3, we further highlight the most popular approaches and mark those that are accessible and functional.

**Table 3:** Summary of the most popular bioinformatics approaches for enhancer identification

Name	Computational method	Highlight	Link	Reference
Heintzman *et al.*	Clustering and correlation of histone marks profiles	High-recognition performance in HeLa	–	[21]
ChromaSig (*)	Identification of specific histone mark motifs and clustering	The method is sensitive enough to capture patterns characterizing different classes of enhancers.	http://bioinformatics-renlab.ucsd.edu/rentrac/ wiki/ChromaSig	[22]
Rye *et al.*	Clustering of profiles	The results indicate that selection of relevant TFs may be sufficient to identify regulatory elements	–	[23]
Won *et. al*.	HMMs	State-of-the-art method suggesting that HMMs are capable of integrating information from multiple histone marks for predicting regulatory elements	http://http/nash.ucsd.edu/chromatin.tar.gz	[54]
Boyle *et al.*	Combination of DHS with TFBSs	Active enhancers usually overlap with open chromatin regions, but not all of the DNA accessible regions correspond to enhancers	–	[25]
ChromHMM (*)	HMMs	State-of-the-art genome annotation method by ENCODE	http://compbio.mit.edu/ChromHMM	[30]
Segway (*)	DBNs	State-of-the-art genome annotation method by ENCODE	http://www.pmgenomics.ca/hoffmanlab/ proj/segway/	[31]
ChroModule	HMMs	Annotated human genome for eight cell lines and improved the AUC compared with state-of-the-art HMM based methods	–	[32]
CSI-ANN (*)	ANNs	Effective combination of ANNs with FDA for FS	http://www.healthcare.uiowa.edu/labs/ tan/CSIANNWebpage.html	[33]
ChromaGenSVM (*)	SVMs	Effective combination of SVMs with GA for optimization and FS	http://sysimm.ifrec.osaka-u.ac.jp/download/ Diego/	[34]
EnhancerFinder	MKL	Functional genomics combined with sequence motifs can accurately identify developmental enhancers	–	[35]
RFECS (*)	RFs	Method less prone to overfitting, which introduces additional novelties on the way enhancer predictions are validated	http://enhancer.ucsd.edu/renlab/RFECS_ enhancer_prediction/	[37]
DEEP (*)	SVMs and ANNs	Novel ensemble-learning-based algorithm with good generalization capabilities in unknown cell lines..	http://cbrc.kaust.edu.sa/deep/	[36]
kmer-SVM (*)	SVMs	Study extensively the enhancer sequence context	http://kmersvm.beerlab.org/	[39]
dREG (*)	SVR	Usage of GRO-seq data combined with regression analysis	https://github.com/Danko-Lab/dREG/	[41]
DELTA (*)	AdaBoost	Introduces the concept of shape features from ChiP-seq data	https://github.com/drlu/delta	[38]
Andersson *et al.* (*)	eRNA expression analysis	Introduces one of the most accurate features for enhancer identification	http://enhancer.binf.ku.dk/enhancers.php	[40]
CoSBI (*)	Bi-clustering	Reports combination of histone marks with high discriminative power for the category of enhancers	http://www.healthcare.uiowa.edu/labs/tan/ CoSBIWebpage.html	[24]

*Note*. With (*), are marked the methods that provide source codes or executable files.

## Identification of enhancers based on clustering of epigenetic profiles, DHS and TFBSs

Over the past years, advances in high-throughput experiments such as ChIP-seq have generated vast amounts of data describing the epigenetic landscape of different human and non-human cells and tissues [55–57]. The produced data characterize profiles of different epigenetic marks, identify or estimate many TFBSs and describe the chromatin accessibility of DNA. Systematic analysis of these data generated global epigenetic maps for different cell lines and tissues and enabled inference of the core principles that characterize different categories of DNA regulatory elements [58]. For example, based on data from ChIP-seq experiments, it is found that active enhancers are frequently associated with H3K27ac, while active and poised enhancers are associated with H3K4me1 [17]. Such information made space for the development of several computational methods for identification of enhancers and other regulatory elements in a cell-line-/tissue-specific context. Essentially, all of the methods that fall into this category initially estimate the profiles (therefore called epigenetic signatures) of histone marks and/or the profile of DHS from different genomic regions. In a later step, these genomic regions are assigned into different regulatory classes via unsupervised learning techniques (e.g. grouping of similar epigenetic profiles) or by the binding fingerprint of enhancer-related TFBSs [59–61].

### Methods based on Clustering of Chromatin Profiles

Typical example of this subcategory is the bioinformatics analysis presented in Heintzman *et al.* [21], which studied the chromatin landscape of promoters and enhancers in HeLa cell line from ENCODE experiments [62]. In the first stage, the analysis revealed that promoters are characterized by H3K4me3, while enhancers are characterized by H3K4me1, but not H3K4me3. In the second stage, the outcome of this analysis served as a basis for developing a two-step algorithm that scans genomic regions from new cell lines and classifies genomic segments as promoters and enhancers based on the similarity of chromatin profiles with existing annotated segments. Although the reported enhancers [21] were derived from a single data set, the main findings have served as a baseline for many subsequent studies for enhancers characterized by the presence of P300-binding sites. Another example is ChromaSig [22] that uses signatures of nine core chromatin marks to generate groups of distinct histone modification profiles that can be further assigned to different classes of regulatory elements. Analysis over HeLa and CD4T cells identified 8 and 16 clusters of chromatin profiles, respectively, that were enriched in enhancer- and promoter-related TFBSs. Overall, ChromaSig is sensitive enough to distinguish different classes of enhancers, and the results are in agreement with the enhancer lists reported by previous studies [21].

Following the above-mentioned concepts, several other methods [23, 24] used diverse data sets and different clustering techniques to identify enhancers. As an example, clustering of TFBS profiles from 67 binding factors and 9 histone marks from ENCODE Gm12878 and K562 cell lines revealed that between those two cell lines, H3K4me1 marker is more frequent in enhancer clusters compared with P300 or H3K27ac [23]. The main outcome of this study indicates that an adequate selection of TFs may be used to identify different regulatory elements in the genome. In another study, the problem of describing more effectively combinatorial histone modification patterns is tackled using a novel algorithm for clustering called CoSBI (Coherent and Shifted Bicluster Identification) [24]. CoSBI follows the concept of coherent bi-clustering applied to 39 chromatin modification maps from CD4T cells [63]. The algorithm reported 843 patterns of core chromatin modification marks that effectively distinguish different regulatory elements, including the category of enhancers.

### Methods based on Chromatin Accessibility and TFBSs

There are several other studies for enhancer recognition that rely mainly on the effective combination of DHS footprints with TFBSs of enhancer-related binding factors like P300 or CREBBP (therefore called CBP) [64, 65]. Here, we highlight the high-resolution identification of DNA regulatory elements in seven lymphoblastoid cell lines and other five human cells/cell lines with diverse characteristics [K562, HeLa, HUVEC, NHEK and embryonic stem cells (ESCs)] [25]. Active enhancers were found to overlap with DHS. Note that not all highly accessible DNA regions correspond to enhancers. To mitigate the above-mentioned limitation, DHS information can also be combined with more advanced algorithms such as CENTIPEDE [26] and Wellington [27] for identifying binding sites of enhancer-related binding factors. We note that TFBSs and ChIP-seq data from histone marks, combined with PGMs and clustering techniques, have been successfully applied to studies of the mouse genome [28, 29]. Finally, an algorithm called Prestige [66] uses histone H3K4me1 profiles from ChIP-seq data, combined with gene expression from RNA-seq, to identify enhancers and associate variations of the enhancer region sequences with diseases through genome-wide association studies.

## Identification based on ML classification methods

Methods of this category reformulate the enhancer identification problem as a binary classification task for predicting enhancer regions as being different from non-enhancer (negative control) regions. So far, SVMs, ANNs, DTs, RFs, PGMs and ensemble techniques have successfully been applied. All these methods have found use in bioinformatics [67–69] and could be applied to enhancer prediction problems [30–38]. We also note that ensemble-learning methods have documented advantages for the class-imbalance problem, which is also present in enhancer identification [69]. Briefly, the class-imbalance problem occurs when the number of samples from the class of interest (e.g. enhancers) differs significantly from the number of samples from other classes (e.g. non-enhancers).

Typically, supervised ML classification systems are combined with feature selection (FS) techniques to extract small sets of features (in our case, histone modification marks and/or sequence characteristics and/or TFBS/binding motifs), which, all together, are capable of maximizing the separation between enhancers and non-enhancers [70, 71]. In addition, a combination of supervised classification systems with global optimization techniques such as Genetic Algorithms (GA) or Simulated Annealing can be used for tuning the model parameters and optimizing several steps of the enhancer recognition process [72].

### Solutions that use PGMs

The methods we survey here are used for genome-wide annotation purposes. In principle, some of these tools [30, 31] segment genomes into intervals and develop PGMs from large numbers of chromatin modifications coming from multiple cell lines and tissues. The identified chromatin states are then grouped and annotated as enhancers, promoters, repressed regions or transcribed regions based on the known functional sites.

The most popular genome-wide annotation tool for genome segmentation in the above-mentioned manner proposed by the ENCODE consortium is ChromHMM [30]. ChromHMM uses a probabilistic model based on a multivariate HMMs. ChromHMM segments the genome into 200 bp intervals, and a single model is trained on data from six available cell lines. Segway [31], on the other hand, is an alternative genome annotation tool based on DBNs. Segway offers a higher-resolution analysis because it annotates the genome for every single base (e.g. has 1 bp resolution). In addition, it trains cell-specific models and is more computationally demanding than ChromHMM.

Although ChromHMM and Segway were developed independently, the ENCODE consortium combined these programs to annotate the human genome in a more comprehensive way. The annotation proposed by Hoffman *et al.* [73] combines the results produced by ChromHMM and Segway with other relevant experimental data such as DHS, FAIRE (Formaldehyde-Assisted Isolation of Regulatory Elements) assays and several ChIP-seq data sets for transcription regulators (e.g. CTCF, POL II, P300) to generate annotation maps for Gm12878, K562, H1, HeLa, HepG2 and HUVEC cell lines. Note that this annotation serves as the baseline annotation proposed by the ENCODE consortium. Specifically, the integrative annotation categorizes enhancers into three states, Enh, EnhF and EnhWF, with Enh representing the class of enhancers with the strongest enrichment of TFBS (therefore called strong enhancers) [73]. Finally, other probabilistic graphical methods for enhancer identification exist, as well as many independent genome annotation tools [32, 54, 74, 75]. Here, we highlight ChroModule [32], which annotated human genome characteristics for eight cell lines and reported higher recognition performance compared with [30] as indicated by the area under curve (AUC).

### Solutions that use ANNs

In particular, CSI-ANN [33] is one of the first enhancer classification systems that rely on an ANN using chromatin signatures as input. Putative enhancers derived from human CD4T cell data from Wang *et al.* [63] based on P300 ChIP-seq peak distal to TSS overlapping with computationally predicted enhancers from PreMod database [76]. The FS component of CSI-ANN, based on Fisher Discriminant Analysis (FDA), reported several histone marks such as H3K4me3, H4Ac and H3, which separate enhancers from background sequences in an optimized way. In terms of recognition performance, CSI-ANN reported higher Positive Predictive Value (PPV) on untreated HeLa cells (maximum PPV of 66.3% based on the overlap of predictions with P300- or DHS- or TRAP220-binding sites) as compared with [21] and [54].

### Solutions that use SVMs

ChromaGenSVM [34] is a typical enhancer classification system that uses SVMs. ChromaGenSVM is trained on HeLa enhancer data (the authors also developed a second model on CD4^+^T cells from [63]) from Heintzman *et al.* [21] using core ChIP-seq histone modification markers. For FS and SVM parameter optimization, ChromaGenSVM uses a global optimization technique based on GA. The optimal ChromaGenSVM model identified histones H3, H3K4me1 and H3K4me3 as the most prominent features for describing enhancers versus the background sequences. In terms of recognition performance, ChromaGenSVM reported PPV ∼90% on CD4^+^T and on untreated HeLa cells achieved comparable PPV with [21], [33] and [54] (maximum PPV of ∼57% based on the overlap of predictions with P300- or DHS- or TRAP220-binding sites).

The idea of integrating diverse data sets from multiple sources to accurately identify developmental enhancers is the main contribution introduced by EnhancerFinder [35]. EnhancerFinder’s underlying classification method is based on the use of Multiple Kernel Learning (MKL), with the training data sets derived from VISTA database [77]. EnhancerFinder also investigates the discriminative power of different data sets and features, concluding that sequence motifs, combined with functional genomics data (e.g. H3K4me1 or P300), are capable of identifying enhancers. This, of course, relates only to a subset of enhancers. In terms of recognition performance, when applied to the entire genome, EnhancerFinder predicted 84 031 developmental enhancers and achieved much higher recognition performance compared with [30] and [31].

To achieve better generalization capabilities in unknown tissues and cell lines, DEEP (Dragon Ensemble Enhancer Predictor) [36] introduces a two-layer classification algorithm based on SVMs and ANNs and training based on data from multiple cell lines and tissues. In its first step, DEEP trains multiple SVM models on data from different cell lines and tissues, which are combined in a second step via an ANN for finally distinguishing enhancers from non-enhancers. DEEP uses putative enhancers from the ENCODE annotation proposed by Hoffman *et al.* [73], actively transcribed enhancers from FANTOM5 (Functional Annotation of the Mammalian Genome) Atlas [40], and a small set of developmental enhancers achieved in VISTA database [77]. An exhaustive search technique applied on the set of 11 core histone modification markers revealed that different ENCODE cell lines are characterized by different optimized sets of histone marks. In these sets, only H3K4me1 characterizes enhancer regions from different cell lines studied in DEEP. In terms of performance, DEEP reported higher PPV compared with [30, 31, 33 and 37] on HeLa and K562 cell lines (PPV was computed based on the overlap of predictions with P300-binding sites or DHS). When considering the number of predicted enhancers that overlap with promoters, DEEP achieved lower or comparable overlap with the competitor methods.

### Solutions that use DTs and RFs

For reducing the effects of class-imbalance between enhancer/non-enhancer samples and eliminating limitations coming from the small size of the training data, RFECS (Random Forest-based Enhancer identification from Chromatin States) [37] introduces a RF-based classification system trained on H1 and IMR90 data from the NIH Epigenome Roadmap project [78]. RFECS introduces additional novelties in the way putative enhancer regions are selected and in the way genome-wide predictions are validated. Overall, RFECS tested on CD4^+^T and H1-hESC cell lines achieves higher true-positive rate and lower false-positive rate compared with state-of-the-art enhancer recognition systems [33, 34, 54] (RFECS achieved true-positive rate of ∼70% and ∼82.5% and false-positive rate of ∼7% and ∼4.9%, respectively). We note that the true-positive rate was measured by the overlap of predictions with DHS-, P300- and CBP-binding sites and the false-positive rate was measured by the overlap of predictions with TSSs as annotated by UCSC Genome Browser. In addition, an out-of-bag FS technique reported histone marks H3K4me3, H3K4me1 and H3K4me2 as the most important features for the enhancer’s recognition problem by this approach. DTs have been successfully applied in another method called DELTA (Distal Enhancer Locating Tool based on AdaBoost) [38]. DELTA is based on the AdaBoost algorithm applied to a set of features characterizing the shape of ChIP-seq peaks of core chromatin markers. In terms of performance, DELTA further improved the prediction accuracy on CD4^+^T and H1-hESC cell lines, achieving a misclassification rate of 2% and 1.6%, respectively.

### Solutions that use classification algorithms to study the enhancer DNA sequence context

The problem of identifying enhancers based solely on sequence characteristics (e.g. motifs or kmers) is tackled in [79]. In another study [51], sequence features capable of discriminating mammalian enhancer sequences from random genomic loci are systematically identified. The proposed ‘kmer-frequency vector' [39], which captures the full set of kmers of varying length (3–10 nucleotides), and its refined version called ‘gapped kmer-vector' [80] were used in SVM models to predict enhancers.

## Identification of enhancers using high-resolution data

The presence of deep sequence data has enabled development of a variety of bioinformatics methods to detect active enhancers and test directly their ability to trigger transcription in messenger RNA (mRNA) promoters. Nowadays several enhancer-testing and *in vivo*-screening methods exist for human, mouse, flies and yeast, such as STARR-seq [44], CRE-seq [45], FIREWACh [46] and several others [47–49], which are surveyed comprehensively in [17].

### Methods based on Enhancer Screening Data

This subcategory of methods describes bioinformatics analyses for investigating mechanisms that trigger regulation activities related to enhancers and promoters, combining several high-throughput data sets, sequence characteristics or TFBSs and more targeted mutation experiments [81, 82]. A typical example is an analysis based on MPRA-derived data (massively parallel reporter assay) from K562 and Hep cell lines that reconfirmed previously published results for cell type specificity of enhancer chromatin states [42]. In a similar fashion, functional testing of computationally predicted enhancers with CRE-seq data in K562 cell line revealed that previously reported chromatin states can distinguish active enhancers from negative samples, but TFBS motifs also have high discriminative power and characterize in a better way the most active enhancer regions [43]. Note that an analysis based on STARR-seq data from Drosophila cells reported interesting mechanistic properties of enhancers and can serve as a paradigm for similar studies in humans [52].

### Identification based on Quantification Analysis of RNA

A popular subcategory identifies enhancer regions using high-throughput techniques that measure the production of RNA based on Cap Analysis of Gene Expression (CAGE) or calculation of transcription rate using Genomic Run-on (GRO-seq). In particular, using bidirectional CAGE tags, over 135 tissues and 241 cell lines were analysed in FANTOM experiments [83]. A total of 43 011 putative enhancer regions that were depleted in CpG islands were reported [40]. The so-called ‘Atlas of actively transcribed enhancers' also reported core differences between enhancers and mRNA promoters, whereas the results complement findings reported by the ENCODE consortium. Note that another CAGE analysis from FANTOM5 data revealed that transcription in enhancer regions is the earliest event that leads to many subsequent transcriptional changes during cellular differentiation [84]. Finally, a high-throughput recognition system called dREG [41] uses GRO-seq data [85] and Support Vector Regression (SVR) to identify and characterize effectively active transcriptional regulatory elements, including the category of enhancers.

## Challenges and obstacles in computational identification of enhancers

Here, we address several challenges and open questions related to the enhancer identification.

### Challenges and open questions

Computational prediction of enhancers does not guarantee that the identified enhancers are real. Because there exists no large, sufficiently comprehensive and experimentally validated enhancer set for humans (or other species), one of the major issues related to enhancer identification is how to assess the correctness of predictions. One possible way of validation is to link the predicted enhancers to their target genes. This, complementary to computational prediction of enhancers, is without a doubt the most difficult challenge. Below, we summarize the most important streams for enhancer target identification, and we discuss relevant sub-problems:

- Enhancers can be located relatively close (e.g. few thousands of bases) or much further away (e.g. hundred thousands of bases) to the genes they affect [86]. Consequently, some methods identify enhancer targets based on their relative location to enhancers (e.g. an enhancer interacts with its neighbouring mRNA promoter). These models are oversimplified because there are no clear distance boundaries for the enhancer–promoter interactions. Some of the existing approaches [87] have defined arbitrary thresholds for the relative location of enhancers and mRNA promoters (e.g. minimum distance 5000 bases and maximum 125 000 bases). Although these approaches are easy to implement, they generate a trade-off between distance threshold and number of true and false positives.
- More sophisticated approaches for identifying enhancer targets can be based on correlated activity of enhancers and mRNA promoters. This category is promising because it is based on cell-line-/tissue-specific information. However, the largest obstacle stems from the limited knowledge about enhancer and mRNA promoter co-activity [40, 65]. One possible solution can be based on the identification of all possible pairs of enhancers and promoters within a predefined distance threshold combined with correlation analysis and representative data sets and markers (e.g. correlated expression activity between eRNAs and target genes or correlated DHS activity) [84]. However, this is also challenging because enhancers and mRNA promoters have many-to-many relationships, meaning that one promoter can be associated with multiple enhancers, and one enhancer can be associated with different promoters. Thus, the problem becomes computationally expensive, and efficient pruning techniques are required to restrict the number of candidate associations between enhancers and promoters.
- The most promising direction for identifying enhancer–promoter associations can be based on chromatin conformation data as captured by 3C/5C [88] or ChIA-PET (Chromatin Interaction Analysis by Paired-End Tag Sequencing) [89]. These data sets can be used to identify associations of enhancers with known mRNA promoters in the three-dimensional space. A typical example of this category is the method introduced in [86], which combines ChIA-PET data with supervised learning based on RFs for linking enhancers to their target genes. With all these methods, there are still areas for improvements, such as noise and bias removal in chromatin conformation data sets or utilization of additional features to link enhancer–promoter associations with regulatory functions with much higher confidence.

Except for the enhancer target identification, identifying the tissue-specific activity of enhancers is another promising area of research. For example, histone modification mark data, DHSs, different TFBSs as derived from ChIP-seq experiments and expression of eRNAs can characterize enhancers in a cell-line-/tissue-specific context. In contrast, sequence characteristics or evolutionary-conserved motifs do not contain sufficient information to describe enhancer activity in different tissues. Consequently, methods that rely solely on ChiP-seq data from histone marks, DHS and/or TFBSs may maximize the enhancer recognition performance in specific cell lines and tissues, but frequently the developed models achieve lower generalization capabilities in unknown cell lines [21, 33–36]. To mitigate this trade-off, mixtures of cell-specific features and sequence characteristics appear to be a promising direction [35, 36].

Another important challenge related to the enhancer identification problem concerns the role of eRNAs in transcription regulation. Recent evidence [90] indicates that many TSSs of eRNAs and protein-coding genes present similar architecture that is differentiated only at the post-transcriptional regulatory layer. Consequently, understanding the functional mechanisms of eRNAs and inferring rules that link eRNA transcription with transcription initiation through mRNA promoters [84] is a question warranting further exploration.

### Obstacles of existing approaches

Many obstacles derive from the input data sets that existing methods use and the fact that an optimal combination of features for describing enhancers across different cell-lines and tissues does not exist. [36]. There are also specific technical limitations introduced by the existing computational solutions.

Regarding the used data sets and features, it is documented that information on evolutionary conservation cannot help much [91] in the prediction of enhancers’ activity because few non-coding elements and motifs appear to be well conserved in other species, and because enhancers are largely tissue specific. On the other hand, ChIP-seq data for histone marks and TFBSs capture cell-line-/tissue-specific information. Using these ChIP-seq data, however, requires a demanding data preprocessing phase. This preprocessing phase usually segments genome into small intervals (e.g. 100or 200 bp), but a clear answer to the optimal way of selecting this interval size does not exist. The step of identifying significant ChIP-seq peaks (therefore called the peak-calling step), as derived from programs like MACS (Model-based Analysis of ChIP-Seq) [92] or SICER (Spatial clustering approach for the identification of ChIP-enriched regions) [93], is sensitive to the selection of parameters, which are usually data set dependent and different among different cellular conditions (e.g. HeLa versus K562). Guidelines about the optimal selection of publicly available peak-calling programs for ChIP-seq data can be found in [94] and [95]. Note that some of the existing approaches for enhancer prediction recommend use of specific ChIP-seq peak-calling programs [34, 37], which represent a limitation because different and possibly better solutions for peak calling could be available in future. Furthermore, ChIP-seq data are not available for many of the existing cell lines and tissues. This represents a real obstacle, as it limits the scope of potential studies that rely on such information. To mitigate this problem, data imputation techniques for histone modification marks have been proposed [96].

Moreover, methods that rely on DHS footprints for finding regulatory elements usually lack specificity between different functional categories (e.g. promoters versus enhancers versus insulators) [97]. In other words, DNA regions with enriched DHS activation are not necessarily enhancers. Also, the identification step of TFBSs is also problematic because not all of the enhancers are marked by the same combination of regulatory proteins or present similar histone modification patterns. This simply means that genomic regions with enrichment in specific histone marks (e.g. H3K4me1) or binding factors (e.g. P300) are not necessarily enhancers. To complicate the problem even more, even the antibodies that are used by ChIP-related experiments may not be always available because enhancers are characterized by different (and maybe unknown) combinations of enhancer co-activators [4]. On the other hand, identification of binding sites based on the Positional Weight Matrices (PWMs) prediction models faces limitations and frequently achieves poor recognition performance [98, 99].

Further, supervised and unsupervised ML methods also face limitations. For the unsupervised clustering of histone mark profiles, rules that have been applied for identifying enhancers are not general enough because different combinations of histone markers and enhancer-related TFBSs characterize enhancers in different cell lines and tissues. This argumentation raises several questions that need to be addressed. For example, to what extent chromatin-defined enhancers in multiple cell lines/tissues have exactly the same chromatin states? Or which cell lines and tissues have exactly the same sets of active enhancers?

In addition, the main challenge that all of the ML-based classification methods face is the selection of high-quality samples to represent adequately the positive (enhancers) and negative classes (non-enhancers). In the absence of a ‘ground truth enhancer' data set, the first ML-based classification systems introduced rules to select enhancer regions for training [33, 34, 37]. The most prominent rule is the selection of DNA segments distal to protein-coding TSSs, characterized by open chromatin as indicated by DHS data that are also enriched in enhancer-related TFBSs (e.g. P300 and/or CBP). For the selection of negative samples, random sequences not annotated as enhancers or promoters are frequently used. An alternative way to generate negative control samples is to shuffle the genomic content of existing enhancer regions (e.g. scrambled enhancers). However, with the recent advances on computational and experimental techniques, the ENCODE integrative annotation [70], the Atlas of actively transcribed enhancers [40], the VISTA enhancer browser [75] and the outcome of individual studies based on enhancer-screening data (similar to those we summarized before) can serve as baseline sources for implementing more reliable ML-based recognition systems [35, 36].

Finally, the class-imbalance problem [36, 37], tuning of classification model parameters (e.g. number of neurons or hidden layers for ANNs or parameter C and gamma for SVMs) [34], overfitting issues, poor generalization capabilities of the developed models in unknown cell lines/tissues and *ad hoc* rules for validating genome-wide predictions of enhancers are some technical problems related to enhancer recognition via ML-based classification systems.

## Enhancer-related resources

In this section, we report available online resources related to enhancers, which include databases, repositories of experimental data, computational tools and other material useful for subsequent enhancer identification studies.

Regarding the enhancer databases, PReMod [76] (http://genomequebec.mcgill.ca/PReMod/) and PEDB (Mammalian Promoter/Enhancer DataBase) [100] (http://promoter.cdb.riken.jp/) are two of the first resources that archived computationally predicted enhancers in human and mouse. Currently, the state-of-the-art database for enhancers is the ‘Human Transcribed Enhancer Atlas' that contains actively transcribed enhancers based on the analysis of eRNA expression [40] (http://enhancer.binf.ku.dk/enhancers.php). Except for the list of human enhancers in multiple tissues and organs, the Atlas contains utilities for downstream analysis, such as TF motif enrichment in enhancer sequences, as well as a selection of enhancers based on expression levels. In addition, all the results are publicly available as flat files or can be visualized in the Genome Browser. On the other hand, VISTA enhancer browser (http://enhancer.lbl.gov/) contains a set of developmental enhancers extremely conserved in mouse and human [77]. This list of developmental enhancers is experimentally validated in mouse [77]. There are also some other enhancer sources that archive enhancers in an integrative way. Examples are dbSUPER (http://bioinfo.au.tsinghua.edu.cn/dbsuper/index.php), which contains 66 033 super enhancer regions predicted [101] from 96 human and 5 mouse tissues, and DENdb (Dragon Enhancer DataBase) [50] (http://www.cbrc.kaust.edu.sa/dendb/), which is the first online repository of putative enhancers, from 15 ENCODE cell lines computationally predicted by five state-of-the-art ML enhancer recognition systems. DENdb also incorporates utilities such as overlap of enhancers with TFBS from ChIP-seq data or predictions of TFBSs obtained by PWM from HOCOMOCO (Homo Sapiens Comprehensive Model Collection) database [102], interactions of enhancers with other genomic loci as captured by chromatin conformation technologies such as 3C/5C or ChIA-PET archived in 4DGenome database [103] (http://4dgenome.int-med.uiowa.edu/) and overlap of enhancers with open chromatin regions via DHS.

## Conclusion

Bioinformatics approaches for enhancer identification are valuable for validating hypotheses and assumptions in gene regulation studies. Here, we went through >30 bioinformatics approaches that have been developed over the past few years. We covered three basic streams of computational methods including: (a) methods that identify DNA regulatory elements via clustering of histone marks profiles, open chromatin information and TFBSs; (b) ML-based classification systems; and (c) bioinformatics analyses based on high-resolution enhancer-screening data sets.

During our review process, we identified and reported limitations and advantages of the existing computational methods. In addition, we summarized a comprehensive list of enhancer resources that include databases for enhancers, data repositories and open-source programs useful for further analyses. A large-scale comparison analysis of the performance of the existing methods may provide meaningful insights about the discriminative capacity of different genomic and epigenetic data sets that feed different computational solutions.

We also commented on some promising areas of research, and we reported challenges that require further investigation. Among them, linking enhancers with their *in vivo* target genes and understanding the role of eRNAs for transcription regulation are among the most challenging topics for future research.

To conclude, we anticipate that our review will complement subsequent gene regulation studies aimed at resolving questions regarding the role of enhancers into cellular transcriptional activities.

Key Points

- Interplay between histone modification profiles, open chromatin information and TFBSs can characterize enhancer regions with increased accuracy in a cell-line-/tissue-specific content.
- Developed models based on SVMs, ANNs, RFs, use with various level of success, features such as binding sites of P300, CBP, TRAP220 proteins, sequence compositional properties, DHS, different chromatin marks.
- The effectiveness of ML models critically depend on the selected set of features, and the most promising solutions use combinations of features deriving from genomic and epigenomic data.
